# Single snare pushing technique: A new bailout technique for retrieving Micra fixed in the tricuspid valve annulus

**DOI:** 10.1002/joa3.70027

**Published:** 2025-02-17

**Authors:** Keisuke Kojima, Atsushi Tanaka, Nozomi Kitade, Hiroshi Ikuta, Junichiro Nishi

**Affiliations:** ^1^ Department of Cardiology Aso Iizuka Hospital Iizuka Japan

**Keywords:** Micra, recapture, snare catheter

## Abstract

When we recapture the Micra system, we capture the body by pulling the tether attached to the delivery catheter and retrieve it, but sometimes it is difficult, and the Micra is dislodged and fixed in the tricuspid valve annulus. Using a single snare catheter from the superior vena cava and pushing the tines changes the orientation of the device and enables recapture.

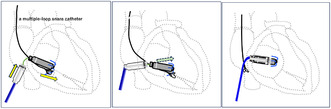

The Micra transcatheter pacing system (Medtronic, Minneapolis, MN, USA) is a common leadless pacemaker fixed to the ventricular septum with four tines. In some cases, the Micra device needs to be repositioned owing to a high pacing threshold for long‐term battery longevity. In these cases, the tether connecting the Micra and the delivery catheter must be retracted into the sheath by traction. However, it is sometimes difficult to capture the tether, and the tether may need to be cut to recapture the Micra using a snare catheter. Here, we report a case in which the Micra fixed to the tricuspid valve annulus (TA) was successfully retrieved into the catheter without cutting the tether using a single snare catheter.

An 83‐year‐old man with bradycardia (35 beats/min) was referred to our hospital. Since he presented with atrial fibrillation and bradycardia and repeatedly experienced cardiac arrest lasting >6 s, each time complaining of chest discomfort, a leadless pacemaker was recommended.

Before the procedure, a temporary pacemaker was inserted into the right jugular vein. After confirming the anatomy of the right atrium (RA) and right ventricle (RV) by injecting contrast into the RA using a pigtail catheter (Figure 1A), the Micra device was implanted into the RV.

**FIGURE 1:**
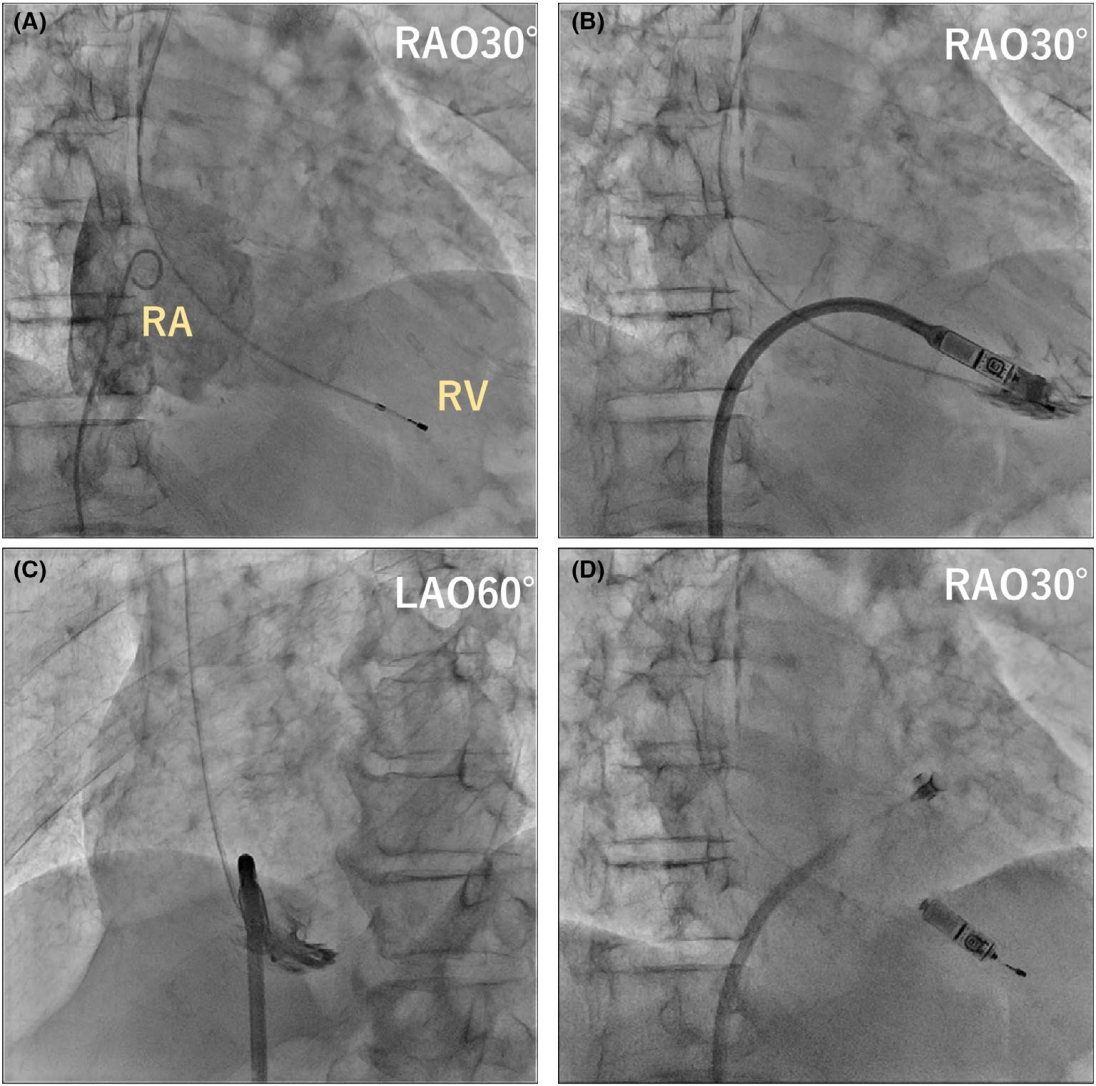
(A) Right atrial and right ventricular angiography with a pigtail catheter before the first attempt. (B, C) Right ventricular angiography showing the location of the Micra at the first attempt; it was located at the apical ventricular septum. (D) The position of the first release of the Micra. LAO, left anterior oblique; RA, right atrium; RAO, right anterior oblique; RV, right ventricle.

We tried the first deployment at the apical interventricular septum (Figure 1B–D), but our attempt failed because there was only one tine attachment to the ventricle, and the pull‐and‐hold test showed that the fixation was insufficient. We decided to recapture the Micra device and reattempt its deployment. However, recapturing it was difficult because of the unfavorable angle between the recapture cone and the device. After prolonged manipulation, the device was dislodged from the myocardial anchorage of the RV, entangled in the tissue near the TA, and could not be moved (Figure 2A). By pulling on the tether, we attempted to change the angle of the Micra while bringing the cone closer, but we were unable to recapture it because the device was fixed in the TA.

**FIGURE 2:**
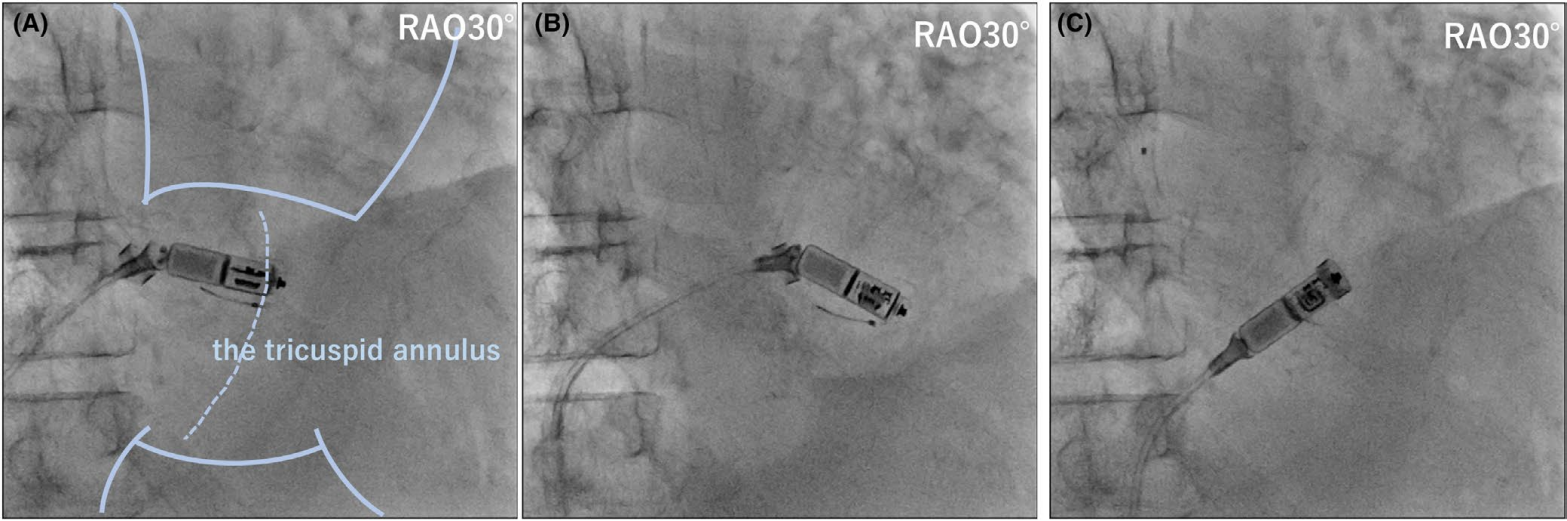
(A) The Micra device was attached to the tricuspid annulus (TA). A multi‐loop snare (a 4–8‐mm loop, EN snare®; Merit Medical Systems, South Jordan, UT, USA) catheter is used to catch the inferior tines from the superior vena cava. (B) We pushed the snare catheter, and the Micra moved; the retrieval head changed direction and became coaxial with the Micra. (C) We successfully recaptured the Micra in the delivery catheter (see Supporting Information Video). RA, right atrium; RAO, right anterior oblique; RV, right ventricle; TA, tricuspid annulus.

We repeatedly pulled the tether while changing the direction and curvature of the delivery sheath; however, it was impossible to recapture the target, regardless of the number of attempts. We hypothesized that some Micra tines were caught and anchored to the tissue surrounding the TA. After extracting a temporary pacemaker, we inserted a multiple‐loop snare (4–8 mm loop, EN snare®; Merit Medical Systems, South Jordan, UT, USA) catheter through the right internal jugular vein. The largest snare catheter that could be inserted into a 5Fr sheath was used, but capturing the Micra body with a tether was not expected; hence, we changed the target to the tines. The moderately bent catheter could easily capture one of the inferior tines. We gradually advanced the Micra using the snare catheter toward the RV, hoping that one of the tines would be released. Then, the Micra moved as it was unfastened (Figure 2B). We loosened the Micra's fixation on the tissue and changed the angle of the retrieval head. After releasing the snare catheter from the tine, we successfully retrieved the Micra into the recapture cone by pulling it closer (Figure 2C). After confirming no obvious damage to the Micra device and ensuring that the tines were not bent or broken, we re‐implanted the same Micra at a higher ventricular septum than the first time (Figure 3). In the second attempt, we confirmed the fixation of two or more tines and a good threshold value (threshold: 1.0 V/0.24 ms, pacing impedance: 520 Ω, R wave sensing: 4.8 mV).

**FIGURE 3:**
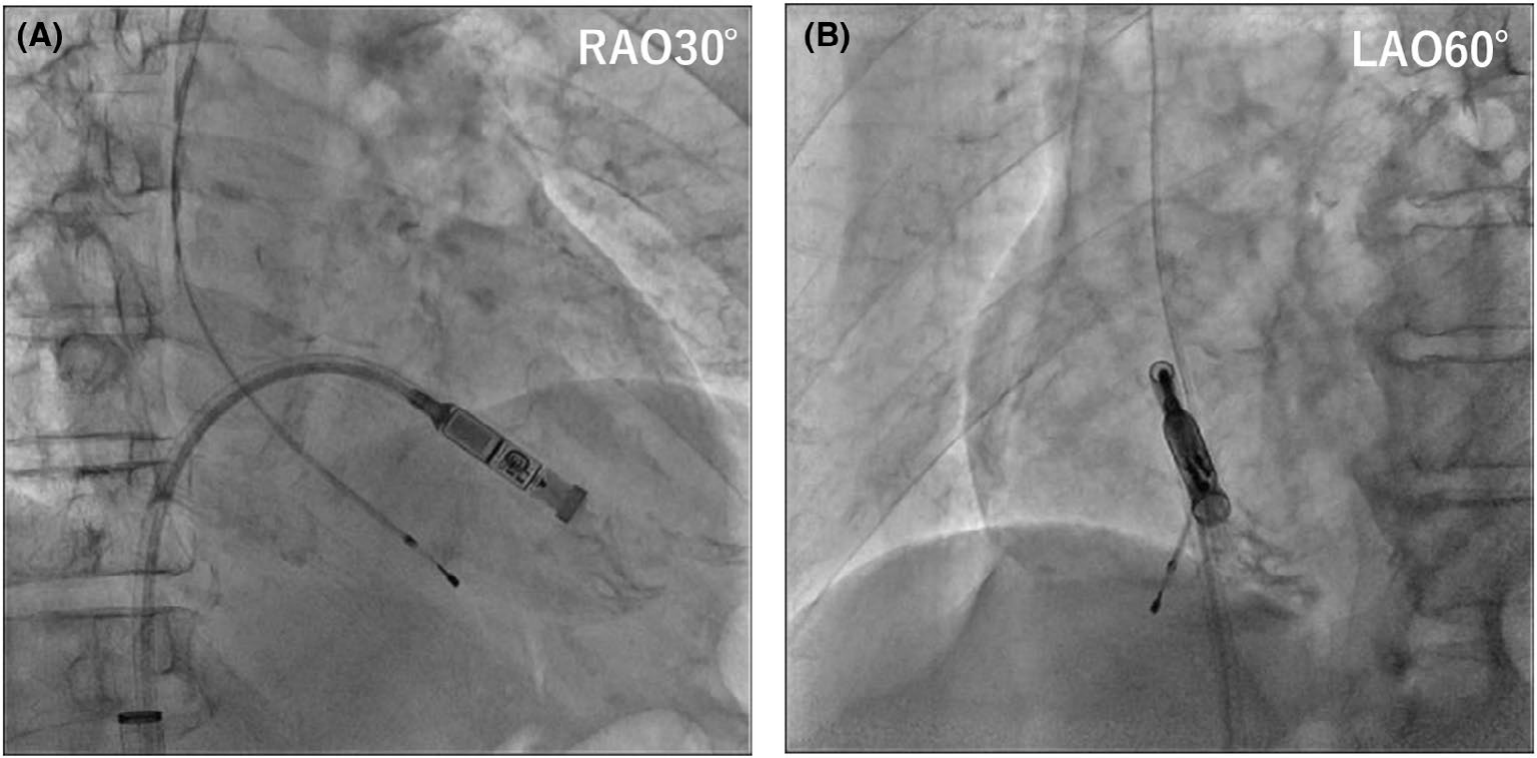
(A, B) Right ventricular angiography shows the location of the Micra device at the second attempt. LAO, left anterior oblique; RAO, right anterior oblique.

No tricuspid valve regurgitation or pericardial effusion was observed on transthoracic echocardiography. The patient was discharged without any complications.

In the Micra system, the operator captures the body by pulling the tether attached to the delivery catheter and retrieving it from the catheter. However, if the angle difference between the Micra in the RV septum (particularly in the apical septum) and the sheath curve is large, recapture may be challenging. In some cases, the Micra was embolized into the pulmonary artery due to a tear in the tether, and some snare catheters were used.^1^, ^2^ In our case, pulling the tether dislodged the Micra without bringing the catheter tip to the retrieval head of the Micra, which caused this issue. The micra, once dislodged from the septum, is thought to be anchored to other tissues in the RV, with various intricate tissues such as tendons, papillary muscles, and trabecular muscles.

Some reports have described using double snare catheters in the superior and inferior vena cava to snare and retrieve the body of the Micra.^3^, ^4^ Others have used a steerable sheath (e.g., Agilis NxT™; Abbott Medical) to capture it.^5^ However, to our knowledge, no report has explained a bailout technique for immediately recapturing the Micra caught in the TA without cutting the tether. According to previous reports, usually, the tether is cut completely, the Micra is retrieved using a double snare catheter, and another Micra is re‐implanted when recapturing is not possible using the existing systems. We successfully retrieved the Micra without cutting the tether and without the need for an additional device using a single snare catheter. Our findings suggest that the Micra fixed in RV tissues can be successfully retrieved by manipulating the catheter in the direction to remove the tether. The single snare catheter can be used to supplement the tine even if it has small loops and can be manipulated to change the fixed orientation of the micra and the angle of the retrieval head by pushing the catheter. We believe that an approach from the superior vena cava rather than from the inferior vena cava is suitable for catching the inferior tines and transmitting the pushing force to the catheter more smoothly. This type of acute bailout technique has not been previously reported.

In conclusion, we report a case of the recapture of the Micra device fixed in the TA. A single snare pushing technique for targeting the tines is able to change the orientation of the device fixed in other tissues and is useful for enabling recapture.

## CONFLICT OF INTEREST STATEMENT

Authors declare no conflict of interests for this article.

## PATIENT CONSENT STATEMENT

Written informed consent was obtained from the patients to publish this report.

## Supporting information


Video S1.

Video S2.

## Data Availability

The data that support the findings of this study are available from the corresponding author upon reasonable request.
